# Factors Associated with Severe Respiratory Support Requirement in Neonates with Gram-Negative Infections: A Retrospective Cohort Study

**DOI:** 10.3390/jcm15145534

**Published:** 2026-07-15

**Authors:** Mihaela Zaharie, Aniko Maria Manea, Mărioara Boia, Nicoleta Lungu, Florina Doandes, Daniela-Eugenia Popescu, Daniela Iacob

**Affiliations:** 1 Department of Neonatology and Puericulture, “Victor Babes” University of Medicine and Pharmacy of Timisoara, 300041 Timișoara, Romania; mihaela.zaharie@umft.ro (M.Z.); boia.marioara@umft.ro (M.B.); lungu.nicoleta@umft.ro (N.L.); doandes.florina@umft.ro (F.D.); popescu.daniela@umft.ro (D.-E.P.); iacob.daniela@umft.ro (D.I.); 2 Neonatology and Preterm Department, “Louis Ţurcanu” Children Emergency Hospital, 300011 Timișoara, Romania; 3 Medici’s MedLife Hospital Timișoara, Ciprian Porumbescu Street No. 9, 300236 Timișoara, Romania; 4 Clinic of Neonatology, “Pius Brinzeu” County Clinical Emergency Hospital, 300723 Timișoara, Romania

**Keywords:** neonatal sepsis, Gram-negative infection, respiratory failure, mechanical ventilation, prematurity, maternal risk factors, neonatal intensive care, respiratory support escalation, risk stratification

## Abstract

**Background:** Gram-negative neonatal infections are associated with severe clinical courses and frequently require respiratory support. However, factors associated with the requirement for invasive mechanical ventilation (IMV) after infection onset remain incompletely characterized. This study evaluated maternal and neonatal variables associated with respiratory severity in neonates with confirmed Gram-negative infections. **Methods**: A retrospective cohort study was conducted in a tertiary neonatal intensive care unit and included 98 neonates with microbiologically confirmed Gram-negative infections between January 2021 and December 2024. The primary outcome was the requirement for IMV during the early NICU course. Maternal, perinatal, and neonatal characteristics were analyzed using univariate and multivariable logistic regression. Discriminative performance was assessed using receiver operating characteristic (ROC) analysis and an exploratory ridge logistic regression model. **Results:** Sixty-five neonates (66.3%) required IMV, while 90 (91.8%) required some form of respiratory support. Ventilated neonates had a lower gestational age than non-ventilated neonates (35 vs. 38 weeks, *p* = 0.02). In univariate analysis, decreasing gestational age was associated with higher odds of IMV (OR 0.87 per week increase, 95% CI 0.77–0.98, *p* = 0.03), while maternal premature rupture of membranes (PROM) was associated with increased ventilation requirements (OR 6.39, 95% CI 1.39–29.36, *p* = 0.02). Cesarean delivery was also associated with higher odds of IMV (OR 2.81, 95% CI 1.19–6.62, *p* = 0.02). In multivariable analysis, neither gestational age (adjusted OR 0.87, 95% CI 0.74–1.01, *p* = 0.07) nor PROM (adjusted OR 2.10, 95% CI 0.65–6.80, *p* = 0.21) remained independently associated with IMV. Gestational age demonstrated the highest discriminative performance for predicting IMV (AUC = 0.73), followed by birth weight (AUC = 0.65). The exploratory ridge model achieved an apparent AUC of 0.76, with an optimism-corrected AUC of 0.70. Mortality occurred in 24 neonates (24.5%), all of whom required IMV. **Conclusions**: Early respiratory severity was common among neonates with Gram-negative infections, with nearly two thirds of infants requiring IMV. Lower gestational age, PROM, and cesarean delivery were associated with greater respiratory support requirements in unadjusted analyses, although these associations were attenuated after multivariable adjustment. The exploratory prediction model demonstrated moderate discriminative performance. Larger multicenter studies are needed to validate these findings and improve early risk stratification.

## 1. Introduction

Neonatal sepsis remains a major cause of morbidity and mortality worldwide, despite advances in perinatal care and antimicrobial therapy [1]. The incidence of neonatal sepsis varies widely across regions, with rates reported to be up to 40-fold higher in middle-income countries than in high-income settings [2]. Among the causative pathogens, Gram-negative bacteria represent a particularly challenging category, often associated with rapid clinical deterioration, systemic inflammatory response, and the requirement for acute organ support [3,4]. In the neonatal intensive care unit (NICU), Gram-negative infections are frequently associated with severe respiratory distress, extended hospitalization, and adverse short-term outcomes [4,5].

Prematurity and low birth weight are well-established determinants of neonatal vulnerability to infection and respiratory morbidity [6]. Immaturity of the immune system, underdeveloped pulmonary structures, and limited physiological reserve collectively contribute to the increased susceptibility of preterm infants to severe infectious diseases [7,8]. Maternal health also plays a critical role, as maternal bacterial colonization has been linked to neonatal infections including sepsis caused by Gram-negative organisms [9]. Maternal and perinatal factors, such as intrapartum infection, maternal fever, and prolonged or suspected rupture of membranes, have been consistently associated with an increased risk of neonatal sepsis [10,11,12,13].

Respiratory involvement is a common early feature of severe neonatal infection, often reflecting the initial extent of clinical deterioration and can range from supplemental oxygen to invasive mechanical ventilation (IMV). Advances in antenatal corticosteroids, respiratory support, and surfactant therapy have reduced mortality from neonatal respiratory distress syndrome to below 10% in high-resource settings, whereas outcomes remain poor in low-income settings where access to such interventions is limited [14]. Non-invasive ventilation strategies are generally preferred over IMV, as they are associated with lower risks of mortality and bronchopulmonary dysplasia [15,16,17]. Neonatal respiratory distress often presents with nonspecific features shared across multiple conditions, requiring clinical history, laboratory evaluation, and imaging for diagnosis, and is frequently complicated by comorbidities such as air leak syndromes, patent ductus arteriosus, pulmonary hypertension, and sepsis [18]. Understanding which infected neonates are more likely to progress to IMV may support anticipatory clinical decision-making, targeted monitoring, and timely escalation of care.

Much of the existing neonatal sepsis literature has focused on infection incidence, pathogen distribution, antimicrobial resistance, or late outcomes such as mortality and length of hospital stay [19,20,21], with comparatively little known about the early clinical trajectory following Gram-negative infection or how maternal risk exposures and neonatal baseline characteristics interact to shape early disease severity. The current study aimed to identify maternal and neonatal variables associated with requirement for IMV as a marker of severe respiratory involvement in a cohort of newborns admitted to a tertiary NICU with confirmed Gram-negative infections. We selected the requirement for IMV as the primary outcome because it represents an objective, unambiguous, and consistently documented threshold of respiratory failure, in contrast to graded respiratory-support categories that are more susceptible to variation in clinician-level escalation thresholds. IMV also marks a clinically actionable point of deterioration that is closely tied to downstream risks, including nosocomial infection and mortality, making it a relevant target for early risk stratification. By focusing on this outcome, the analysis examines which host factors are linked to early disease progression in infected neonates.

## 2. Materials and Methods

### 2.1. Study Design and Setting

This retrospective observational cohort study was conducted in a tertiary neonatal intensive care unit. Medical records were reviewed to identify neonates diagnosed with Gram-negative infections during the study period (January 2021–December 2024). The aim of the study was to evaluate maternal and early neonatal factors associated with early clinical severity, with particular emphasis on severe respiratory support requirement during the initial NICU course.

### 2.2. Study Population

All neonates admitted to the NICU with a documented Gram-negative infection were eligible for inclusion. Gram-negative infection was defined based on microbiological confirmation from clinically relevant specimens (blood cultures, urine cultures, and tracheobronchial aspirates). Neonates with incomplete maternal or neonatal clinical data were excluded; a total of 12 infants met this criterion, resulting in a final cohort of 98 neonates Because microbiological confirmation of Gram-negative infection frequently occurred after the onset of clinical illness, respiratory support variables were recorded over the early NICU course rather than anchored to a single confirmation timepoint.

### 2.3. Data Collection

Data were extracted retrospectively from electronic medical records using a standardized approach. Maternal and perinatal information included documentation of maternal fever during the peripartum period, premature rupture of membranes (PROM), prenatal antibiotic treatment, and mode of delivery. Maternal PROM was recorded as a documented obstetric diagnosis based on medical records. Although PROM presence was confirmed, precise rupture-to-delivery intervals were not consistently available; therefore, PROM duration could not be operationalized using standardized temporal thresholds and was analyzed as a binary exposure variable.

Neonatal data included sex, gestational age at birth expressed in completed weeks, and birth weight measured in grams. Early neonatal clinical variables focused on respiratory status and included the requirement for respiratory support, the type of respiratory support administered, and the need for IMV. Surfactant administration was also recorded.

### 2.4. Definitions

Respiratory support was defined as the use of free-flow oxygen therapy (via nasal cannula or oxygen hood), high-flow nasal cannula (HFNC, Optiflow system, Fisher & Paykel Healthcare, Auckland, New Zealand), non-invasive ventilation, or IMV. Respiratory severity was characterized according to the highest level of support required during the early NICU course.

IMV was defined as the use of mechanical ventilation modes, including volume-controlled ventilation or synchronized intermittent mandatory ventilation (VM/SIMV).

Surfactant administration was recorded as an adjunct marker of underlying pulmonary immaturity and respiratory disease severity and analyzed separately from infection-related severity. It was not included in the definition of respiratory support.

For severity classification, respiratory modalities were categorized according to the highest level of support required during the early NICU course; therefore, neonates who progressed from non-invasive to invasive ventilation were classified in the invasive ventilation group. The early NICU course was defined as the initial hospitalization period from admission to clinical stabilization or escalation to maximal respiratory support.

Early clinical severity was primarily assessed by the requirement for IMV during the early NICU course.

Given the moderate sample size and the high prevalence of IMV in the cohort, respiratory severity was operationalized primarily as a binary outcome (presence vs. absence of invasive ventilation) for regression analyses, although support intensity was also reported descriptively as a graded measure. Because some neonates were already receiving respiratory support at the time of microbiological diagnosis, respiratory severity was assessed according to the highest level of respiratory support required during the subsequent clinical course following infection recognition.

### 2.5. Outcomes

The primary outcome was the requirement for IMV at any point during the early NICU course. IMV was analyzed as a binary variable (present vs. absent) and was used as the primary marker of early respiratory severity. Within this outcome, neonates who required IMV only after microbiological confirmation (who escalated from a lower level of respiratory support) are considered to reflect early respiratory deterioration in the strict sense, whereas neonates already receiving IMV at the time of microbiological confirmation are considered to reflect baseline respiratory severity rather than deterioration; both subgroups are reported also separately in the Results.

Secondary outcomes included mortality, length of hospital stay, duration of invasive mechanical ventilation and the distribution of respiratory support intensity. Respiratory support intensity was classified according to the highest level of respiratory assistance required during the early clinical course, ranging from oxygen supplementation and high-flow nasal cannula support to invasive mechanical ventilation.

Microbiological species identification was described descriptively but were not included in predictive modeling analyses because of the limited sample size.

### 2.6. Statistical Analysis

Continuous variables were assessed for normality and are presented as medians with interquartile ranges. Categorical variables are reported as frequencies and percentages. Comparisons between ventilated and non-ventilated neonates were performed using the Mann–Whitney U test for continuous variables and Fisher’s exact test for categorical variables. Respiratory modalities were not modeled as separate ordinal categories due to sample size constraints and the high prevalence of invasive ventilation. Comparisons of mortality and length of hospital stay between ventilated and non-ventilated neonates were performed using Fisher’s exact test and the Mann–Whitney U test, respectively. Duration of invasive mechanical ventilation was summarized descriptively among ventilated neonates.

Univariate logistic regression analyses were conducted to explore associations between selected maternal and neonatal variables and the need for IMV. Variables considered for the standard multivariable model were selected a priori based on clinical relevance and univariate trends. Given 65 IMV events in the cohort, a conservative events-per-variable threshold of approximately 10 was applied to the standard multivariable model, which limited the number of retained predictors to two (gestational age and maternal PROM), selected based on their established association with prematurity-related vulnerability and perinatal infectious exposure, in order to minimize overfitting.

Association between causative pathogen and IMV requirement was assessed using the chi-square test of independence, with pairwise comparisons by Fisher’s exact test.

Results are reported as odds ratios with corresponding 95% CI. A two-sided *p*-value below 0.05 was considered statistically significant. Given the limited events-per-variable ratio, penalized logistic regression (ridge) was employed to reduce overfitting risk and improve model stability under small-sample conditions.

An exploratory prediction model for IMV (MV/SIMV) was developed using penalized logistic regression (ridge). Candidate predictors were selected a priori based on clinical availability at presentation (gestational age, birth weight, PROM, maternal fever, prenatal treatment, delivery mode, and sex); the higher candidate-to-event ratio was considered acceptable for the exploratory ridge model given its penalization. The ridge penalty (lambda) was selected via 10-fold cross-validation. Discrimination was assessed by the area under the ROC curve (AUC). Internal validation was performed using bootstrap resampling to estimate optimism-corrected AUC.

Model calibration was assessed alongside discrimination. Neonates were grouped into quartiles of predicted probability from the ridge model, and observed IMV frequency was plotted against mean predicted probability within each quartile. Calibration slope and intercept were estimated by regressing the observed outcome on the model’s linear predictor. The Brier score was calculated as the mean squared error between predicted probabilities and observed outcomes. Calibration slope was additionally corrected for optimism using the same bootstrap resampling procedure applied to the AUC.

## 3. Results

### 3.1. Study Population and Baseline Characteristics

A total of 98 neonates with documented Gram-negative infections were analyzed. Most infants experienced a severe early respiratory course, with approximately two thirds of the cohort requiring IMV (65/98), while the remaining 33 neonates did not require intubation.

The cohort consisted predominantly of preterm infants, with a median gestational age of 36 [IQR 33–37]. Cesarean section accounted for half of all births. PROM was documented in approximately one quarter of cases, whereas intrapartum fever was rare. Prenatal antibiotic exposure was documented in fewer than one in ten pregnancies. In the overall cohort, the median Apgar score was 8 at both 1 and 5 min. Respiratory distress syndrome was present in 46 neonates (46.9%), while surfactant therapy was administered in 10 cases (10.2%).

### 3.2. Comparison Between Ventilated and Non-Ventilated Neonates

Neonates who required IMV were born at a significantly earlier gestational age than those managed without intubation, with a median gestational age of 35.0 weeks compared with 38.0 weeks in non-ventilated infants.

Maternal and perinatal risk factors tended to be more frequently identified among neonates with more severe respiratory courses. PROM was more common in ventilated neonates than in those who did not require invasive support. Maternal fever was recorded only among ventilated cases, whereas the proportion of cesarean deliveries was significantly higher in the IMV group. The use of surfactant therapy did not differ meaningfully between ventilated and non-ventilated neonates.

A comparison of baseline maternal, perinatal, and neonatal characteristics according to mechanical ventilation status is provided in Table 1.

The most frequently identified Gram-negative organism was *Escherichia coli* (53.1%), followed by *Klebsiella* spp. (25.5%) and *Serratia* spp. (21.4%).

### 3.3. Respiratory Support Use and Severity Distribution

Respiratory support was required in 90 of 98 neonates (91.8%) during the early NICU course, whereas only eight infants (8.2%) were managed without any form of respiratory assistance. When respiratory interventions were classified according to the highest level of support required, IMV was the predominant modality, affecting 65 neonates (66.3%). Lower-intensity respiratory support was less common, with non-invasive ventilation (NIV) representing the highest level of support in 11 neonates (11.2%), oxygen therapy alone administered in 10 neonates (10.2%), and high-flow nasal cannula (HFNC) support used in four neonates (4.1%).

Of the 65 neonates who required IMV, 48 escalated to invasive ventilation from a lower level of respiratory support after microbiological confirmation of infection, whereas 17 were already receiving IMV at the time of confirmation. Because the primary outcome was defined as the requirement for IMV (present vs. absent), both subgroups were analyzed together in the primary analyses.

Overall, NIV was used during the clinical course of 32 neonates (32.7%). Among these, 21 infants subsequently required escalation to IVM, whereas 11 were managed without intubation.

### 3.4. Gestational Age Stratification and Severity Patterns

To further examine the relationship between prematurity and early clinical severity, neonates were stratified into three gestational age categories: <32 weeks, 32–36 weeks, and ≥37 weeks. The requirement for IMV varied significantly across these strata (χ^2^ *p* < 0.001), with the highest rates observed among the most premature infants. Mechanical ventilation was required in 19 of 24 neonates (79.2%) born before 32 weeks, increased to 34 of 38 neonates (89.5%) in the 32–36-week group, and declined to 12 of 36 neonates (33.3%) among those born at term.

A similar gradient was observed for PROM, which was most frequent among extremely preterm neonates (11/24, 45.8%) and progressively less common with advancing gestational age (10/38, 26.3% in the 32–36-week group and 2/36, 5.6% in term neonates).

### 3.5. Univariate Predictors of Invasive Mechanical Ventilation

In univariate logistic regression analyses (Table 2), decreasing gestational age was associated with higher odds of invasive mechanical ventilation (OR per 1-week increase = 0.87, 95% CI: 0.77–0.98, *p* = 0.03). A similar directional relationship was observed for birth weight, with increasing weight showing a non-significant protective trend (OR per 100 g increase = 0.95, 95% CI: 0.90–1.01, *p* = 0.08).

Maternal PROM was associated with substantially increased odds of requiring invasive mechanical ventilation (OR = 6.39, 95% CI: 1.39–29.36, *p* = 0.02). Cesarean delivery was also associated with higher odds of ventilation requirement (OR = 2.81, 95% CI: 1.19–6.62, *p* = 0.02). In contrast, female sex, prenatal antibiotic exposure, and surfactant administration showed no significant associations with ventilation requirement.

### 3.6. Multivariable and Logistic Regression Analysis

A multivariable logistic regression model was subsequently constructed incorporating gestational age and maternal PROM, selected a priori based on their clinical relevance. After adjustment, neither gestational age (adjusted OR = 0.87, 95% CI: 0.74–1.01, *p* = 0.07) nor maternal PROM (adjusted OR = 2.10, 95% CI: 0.65–6.80, *p* = 0.21) remained independently associated with the need for invasive mechanical ventilation (Table 3). Although both variables retained effect estimates in the same direction as observed in the univariate analyses, the associations were attenuated after adjustment and no longer reached statistical significance. These findings suggest partial overlap between prematurity and maternal PROM as contributors to respiratory severity in this cohort.

**Figure 1:**
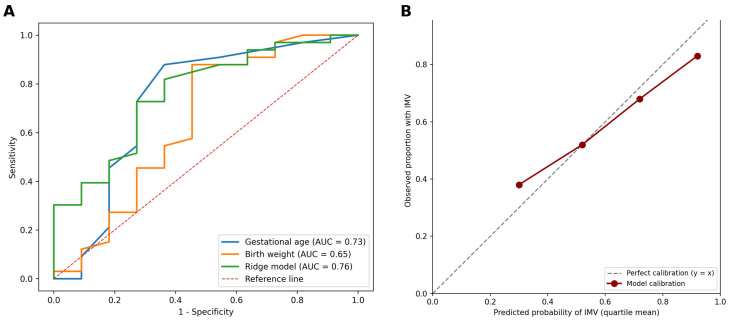
Discrimination and calibration of the exploratory ridge logistic regression model for predicting IMV. (**A**) Receiver operating characteristic (ROC) curves for gestational age, birth weight, and the ridge model; discrimination was quantified by the area under the curve (AUC), with optimism-corrected estimates obtained by bootstrap resampling. (**B**) Calibration plot: mean predicted probabilities (grouped by quartile) versus observed IMV frequencies; the dashed line indicates perfect calibration (y = x).

ROC analysis demonstrated that gestational age showed the highest discriminative ability for predicting invasive mechanical ventilation (AUC = 0.73), followed by birth weight (AUC = 0.65). The exploratory ridge logistic regression model achieved an apparent AUC of 0.76; however, bootstrap internal validation yielded an optimism-corrected AUC of 0.70, indicating moderate discriminative ability and reasonable model stability (Figure 1A).

Across quartiles of predicted risk, mean predicted probabilities of IMV were 0.30, 0.52, 0.72, and 0.92, compared with observed frequencies of 0.38, 0.52, 0.68, and 0.83, respectively (Figure 1B). This pattern indicated mild underprediction at lower predicted risk and overprediction at higher predicted risk. The calibration slope was 0.73 and the calibration intercept was 0.15, consistent with modest overfitting of the apparent model. The Brier score was 0.19. After optimism correction, using the same bootstrap resamples applied to the AUC, the calibration slope decreased further to approximately 0.67, mirroring the shrinkage observed between the apparent (0.76) and optimism-corrected (0.70) AUC.

### 3.7. Clinical Outcomes

Mortality occurred in 24 of 98 neonates (24.5%) and was observed exclusively among infants requiring IMV (24 of 65, 36.9%), whereas no deaths occurred in the non-ventilated group (*p* < 0.001). Ventilated neonates also had a longer median duration of hospitalization (32.0 vs. 23.0 days), although this difference did not reach statistical significance (*p* = 0.18). Among ventilated infants, the median duration of mechanical ventilation was 19 days (IQR 8–32).

IMV requirement did not differ significantly across the three most frequently isolated pathogens (*E. coli* 34/52, *Klebsiella* spp. 19/25, *Serratia* spp. 12/21; *p* = 0.39). No significant differences emerged in pairwise comparisons between any pathogen pair (*E. coli* vs. *Klebsiella*: OR = 0.60, *p* = 0.44; *E. coli* vs. *Serratia*: OR = 1.42, *p* = 0.60; *Klebsiella* vs. *Serratia*: OR = 2.38, *p* = 0.22). Within the limits of the available sample size, early respiratory severity was not strongly pathogen-specific. Although the study was not powered for formal comparisons between pathogen groups, descriptive analyses suggested high rates of invasive mechanical ventilation and substantial mortality across all major organisms.

Pathogen-specific clinical characteristics and outcomes are summarized in Table 4.

## 4. Discussion

In this study, respiratory support requirements were substantial overall, and early respiratory deterioration, defined as an escalation to IMV after microbiological confirmation, occurred in nearly half of the cohort, while a further subset of neonates was already critically ill and receiving IMV at the time of microbiological confirmation. In descriptive and univariate analyses, prematurity was consistently linked to higher respiratory severity; however, after multivariable adjustment, this association was no longer statistically significant. The current study expands on previous research by assessing early clinical trajectories within a population already infected with Gram-negative bacteria, rather than focusing solely on infection risk or pathogen distribution.

Premature newborns with low birth weight represent a particularly vulnerable group in the context of neonatal sepsis, due to the combined impact of pulmonary immaturity, reduced physiological reserve, and an underdeveloped immune response, all of which make them more susceptible to severe infection and early clinical deterioration [22,23,24]. However, previous investigations focused on heterogeneous newborn populations, microbiological features or late outcomes, rather than early respiratory trajectories in infected neonates specifically [25,26].

Maternal risk factors were more frequently identified in neonates requiring more intensive respiratory support. In the present cohort, both PROM and cesarean delivery were associated with an increased likelihood of invasive mechanical ventilation in univariate analyses, whereas these associations were attenuated after adjustment for gestational age. This finding suggests that part of the observed effect may be mediated through prematurity-related vulnerability rather than representing an independent factor associated with respiratory severity. Prior studies have demonstrated the role of maternal membrane rupture and infection in increasing neonatal sepsis risk [27]; however, their influence on early clinical severity once infection is established remains less clearly defined.

Respiratory compromise is among the earliest and most clinically relevant manifestations of neonatal sepsis [6,28,29], with prior studies identifying prematurity, mechanical ventilation, low Apgar scores and low birth weight as key determinants of severity and adverse outcomes [22,30,31]. Our findings are consistent with previous reports demonstrating an association between mechanical ventilation and adverse outcomes in neonatal sepsis [32]. In a cohort of 238 neonates with culture-confirmed sepsis, overall mortality reached 45.4%, and mechanical ventilation was identified as a significant factor associated with death [33].

Previous studies have also explored respiratory complications in neonatal sepsis, including the development of acute respiratory distress syndrome as a severe manifestation of early-onset infection. In a large cohort study, low birth weight and markers of systemic illness were identified as independent predictors of acute respiratory distress syndrome in neonates with sepsis [34]. The study of Duyen et al. reported a predominance of Gram-negative pathogens in neonatal sepsis, with Klebsiella pneumoniae as the most frequent organism, and described respiratory symptoms as a common clinical manifestation. Although their analysis focused on pathogen distribution and clinical presentation rather than respiratory severity, their findings support the frequent association between Gram-negative neonatal sepsis and respiratory compromise [35]. In a cohort of neonates with Gram-negative late-onset sepsis, respiratory distress was reported in more than half of cases, alongside other manifestations such as apnea and seizures, with Klebsiella and Acinetobacter species identified as predominant pathogens [36].

Although respiratory escalation was used as a pragmatic severity marker, the contribution of prematurity-related respiratory pathology cannot be completely separated from infection-related deterioration. The high prevalence of Gram-negative organisms in ventilator-associated pneumonia among neonates emphasizes the clinical relevance of early respiratory deterioration, as prolonged mechanical ventilation may further increase exposure to severe nosocomial infections [37].

Although several clinically relevant variables demonstrated significant associations with ventilation requirement in univariate analyses, these relationships were attenuated after multivariable adjustment. Gestational age is a well-established factor associated with respiratory support need independent of infection status, and this pattern was evident in the present cohort, where it showed the strongest univariate association with IMV and the highest discriminative performance of any single variable. Because PROM and cesarean delivery are themselves associated with gestational age, PROM often precipitating preterm delivery, and cesarean delivery being more common in complicated preterm births, their univariate associations with IMV are plausibly mediated, at least in part, through prematurity rather than reflecting independent infection-severity pathways. No variable, including gestational age itself, remained independently associated with IMV after multivariable adjustment. In a cohort with a high event rate (66%), this null adjusted result should itself be considered informative: it suggests that requirement for IVM in neonates already infected with Gram-negative organisms is probably driven by a combination of overlapping, correlated factors that this sample was not powered to disentangle individually, rather than by any single association. The observed effect sizes and consistent directional trends remain clinically notable despite this limitation. Accordingly, these findings should be interpreted cautiously and considered hypothesis-generating and supportive of further investigation in larger, prospective cohorts adequately powered to separate the contributions of prematurity from infection-specific severity.

Early respiratory support trajectories represent measurable markers of infection severity and host vulnerability. Although the exploratory model showed moderate internally validated performance, it is not suitable for clinical use in its current form; rather, it illustrates the potential of linking routine maternal–neonatal variables with escalation-based severity outcomes to inform future risk stratification after external validation.

### 4.1. Perspectives

These respiratory support trajectories reflect clinically measurable system-level responses to infection severity and host vulnerability. The escalation from supplemental oxygen to non-invasive and invasive ventilation reflects the imbalance between respiratory demand and the neonate’s compensatory capacity during systemic infection. Quantifying these escalation patterns may help translate bedside clinical observations into structured descriptors of disease severity that can be explored in predictive modeling contexts. Respiratory support requirements function not only as therapeutic interventions but also as real-time physiologic outputs of disease progression.

Future studies incorporating maternal risk factors, gestational age, inflammatory biomarkers, and respiratory support requirements may contribute to improved early risk assessment and clinical monitoring of neonates with Gram-negative infections. Such approaches could support resource allocation, ventilatory strategy optimization, and anticipatory NICU management. Moreover, the evaluation of clinical severity trajectories may support future research focused on improving early severity assessment during the infectious course.

### 4.2. Limitations

This study has several limitations that should be considered when interpreting the findings. First, its retrospective design inherently limits control over data completeness, exposure measurement, and temporal standardization of clinical interventions. Although clinical and microbiological data were systematically extracted, retrospective ascertainment may introduce information bias and limit the granularity of physiologic and laboratory variables available for analysis. Second, the single-center setting and the modest cohort size constrained statistical power, particularly for multivariable modeling. The tertiary referral nature of the institution further shapes cohort composition and results in a population enriched for more severe cases, as reflected by the high rate of IMV. Additionally, because inclusion required microbiological confirmation, the cohort represents a selected population with the most severe, culture-positive Gram-negative infections. This selection contributes to the high rate of invasive ventilation observed and limits generalizability to the broader spectrum of neonatal sepsis, including milder or culture-negative presentations. Microbiological stratification was not incorporated, and several available variables (inflammatory markers, Apgar score, antenatal corticosteroids, RDS) were excluded from multivariable analysis to preserve parsimony; others were not consistently available. Additionally, while respiratory support duration and escalation trajectories were recorded, the present analysis focused on the presence and maximal level of support rather than time-dependent respiratory outcomes. As an observational cohort without a non-infected comparison group, this design does not permit causal inference regarding whether Gram-negative infection drives respiratory deterioration, nor can it disentangle the respiratory burden attributable to infection from that attributable to prematurity itself. Although IMV requirement remained substantial among term and late-preterm infected neonates, arguing against prematurity as the sole determinant, a formal separation of these contributions would require a matched non-infected control group or a mediation analysis, which the present data do not support. This represents an important direction for future work Finally, although exploratory predictive modeling was performed, the limited sample size and high prevalence of invasive ventilation increase the risk of optimism and restrict the clinical transportability of the model. External validation in larger, multicenter neonatal cohorts will be required before any risk stratification framework derived from these data can be considered for implementation.

## 5. Conclusions

Neonates with Gram-negative infections frequently experienced substantial respiratory compromise, with IMV required in two thirds of cases. Lower gestational age, maternal PROM, and cesarean delivery were associated with an increased likelihood of IMV only in unadjusted analyses, and these associations should be regarded as descriptive and hypothesis-generating rather than as evidence of independent risk factors. Among the variables assessed, gestational age showed the highest univariate discriminative performance (AUC = 0.73), consistent with its established association with respiratory support need, though this does not establish it as a validated independent predictor in this cohort. In multivariable analysis, no evaluated maternal or neonatal variable, including gestational age, remained independently associated with the need for IMV. Mortality occurred exclusively among neonates requiring invasive mechanical ventilation, underscoring the close relationship between severe respiratory deterioration and adverse clinical outcomes. The exploratory prediction model demonstrated moderate discriminative performance after internal validation, indicating that routinely available maternal and neonatal characteristics may contribute to early respiratory risk assessment. Larger multicenter studies are warranted to validate these findings, refine risk stratification approaches, and improve the early identification of neonates at risk of severe respiratory deterioration following Gram-negative infection.

## Figures and Tables

**Table 1:** Baseline maternal, perinatal, and neonatal characteristics stratified by the need for IMV.

Variable	No IMV (*n* = 33)	IMV (*n* = 65)	*p*-Value
Gestational age (weeks), median [IQR]	38 [36–39]	35 [32–37]	0.02
Birth weight (g), median [IQR]	3000 [2260–3500]	2570 [1900–2840]	0.15
Female sex, *n* (%)	4 (12.1)	5 (7.7)	0.48
Cesarean delivery, *n* (%)	11 (33.3)	38 (58.5)	0.03
PROM, *n* (%)	2 (6.1)	19 (29.2)	0.009
Maternal fever, *n* (%)	0 (0.0)	4 (6.2)	0.30
Prenatal antibiotics, *n* (%)	2 (6.1)	6 (9.2)	0.71
Apgar 1 min, median [IQR]	8 [6–8]	8 [6–8]	0.50
Apgar 5 min, median	9 [8–10]	8 [8–9]	0.33
RDS, *n* (%)	12 (36.4)	34 (52.3)	0.20
Surfactant administration, *n* (%)	2 (6.1)	8 (12.3)	0.49

Abbreviations: g—grams; IQR—interquartile range; IMV—invasive mechanical ventilation; *n*—number; PROM—premature rupture of membranes; RDS—respiratory distress syndrome. Continuous variables were compared using the Mann–Whitney U test; categorical variables were compared using Fisher’s exact test.

**Table 2:** Univariate logistic regression analysis of predictors of invasive mechanical ventilation.

Predictor	OR	95% CI	*p*-Value
Gestational age (per 1 week)	0.87	0.77–0.98	0.03
Birth weight (per 100 g)	0.95	0.90–1.01	0.08
PROM (yes vs. no)	6.39	1.39–29.36	0.02
Cesarean delivery (yes vs. no)	2.81	1.19–6.62	0.02
Female sex (yes vs. no)	0.60	0.15–2.34	0.46
Prenatal antibiotics (yes vs. no)	1.57	0.30–8.24	0.60
Surfactant (yes vs. no)	2.18	0.43–11.10	0.35

Abbreviations: CI—confidence interval; OR—odds ratio.

**Table 3:** Multivariable logistic regression analysis.

Predictor	Adjusted OR	95% CI	*p*-Value
Gestational age (per 1 week)	0.87	0.74–1.01	0.07
PROM (yes vs. no)	2.10	0.65–6.80	0.21

**Table 4:** Clinical characteristics and outcomes stratified by the most frequently isolated Gram-negative pathogens.

Pathogen	*n* (%)	GA (Weeks), Median [IQR]	IMV *n* (%)	LOS (Days), Median [IQR]	Mortality *n* (%)
*Escherichia coli*	52 (53.1)	36 [33–38]	34 (65.4)	28 [18–42]	11 (21.2)
*Klebsiella* spp.	25 (25.5)	34 [31–36]	19 (76.0)	35 [25–48]	8 (32.0)
*Serratia* spp.	21 (21.4)	35 [32–37]	12 (57.1)	30 [20–39]	5 (23.8)
Total	98	36 [33–37]	65 (66.3)	27 [19–42]	24 (24.5)

## Data Availability

Data is available on request from the corresponding author.
